# Retinoic Acid Induces Differentiation of Mouse F9 Embryonic Carcinoma Cell by Modulating the miR-485 Targeting of Abhd2

**DOI:** 10.3390/ijms20092071

**Published:** 2019-04-26

**Authors:** Mengying Yu, Lei Zhang, Yingxiang Liu, Defu Liu, Zekun Guo

**Affiliations:** Key Laboratory of Animal Biotechnology, Ministry of Agriculture, Northwest A&F University, Yangling 712100, China; woshihaokkk@126.com (M.Y.); masterzlei@163.com (L.Z.); LYXLY1024@163.com (Y.L.); nwsuaf_liudefu@163.com (D.L.)

**Keywords:** retinoic acid, miR-485, Abhd2, Erk1/2, differentiation

## Abstract

Retinoic acid (RA) plays a key role in pluripotent cell differentiation. In F9 embryonic carcinoma cells, RA can induce differentiation towards somatic lineages via the Ras-extracellular signal-regulated kinase (Ras/Erk) pathway, but the mechanism through which it induces the Erk1/2 phosphorylation is unclear. Here, we show that miR-485 is a positive regulator that targets α/β-hydrolase domain-containing protein 2 (Abhd2), which can result in Erk1/2 phosphorylation and triggers differentiation. RA up-regulates miR-485 and concurrently down-regulates Abhd2. We verified that Abhd2 is targeted by miR-485 and they both can influence the phosphorylation of Erk1/2. In summary, RA can mediate cell differentiation by phosphorylating Erk1/2 via miR-485 and Abhd2.

## 1. Introduction

The ability to differentiate into all cell types is an important characteristic of embryonic stem cells (ESCs) that can be used for the treatment of diseases [1,2]. Before ESCs can be used therapeutically, it is important to understand their differentiation mechanisms. The F9 embryonic carcinoma cell line (F9 ECs), which is derived from a mouse testicular teratoma and originates from pluripotent germ cells, has been used as a model for differentiation [3,4] as it has a self-renewal capacity and maintains an undifferentiated stage. The two types of cells (ESCs and F9 ECs) share many similar mechanisms [3,4] and the differentiation of both can be induced by retinoic acid (RA) [5,6,7] in a process that involves multiple pathways [8,9,10].

Previous reports have suggested that endogenous small-molecule signaling pathways can maintain the pluripotency of mouse embryonic stem cells (mESCs) [11,12] while a medium containing two inhibitors (2i), CHIR99021 (CHIR) and PD0325901 (PD), can support the pluripotency of mESCs. CHIR inhibits glycogen synthase kinase-3 and regulates canonical Wnt/β-catenin signaling to stimulate the self-renewal of ESCs [13] and PD is an inhibitor that represses mitogen-activated protein kinase (MAPK) and Extracellular signal-regulated kinase (Erk) signaling, and thus stabilizes Nanog (since Erk1/2 signaling negatively regulates Nanog transactivation). The suppression of Erk signaling promotes the self-renewal of mESCs [14,15,16]. PD is also one of the inhibitors that inhibits the enhancement of Erk1/2 phosphorylation by fibroblast growth factor-4 (FGF4) stimulation in mESCs, which occurs through the mitogen-activated protein kinase and mitogen-activated protein kinase (MAPK/Erk) pathway [17,18]. It can restrict the differentiation of ESCs by inactivating MAPK/Erk and in our previous study, we showed that PD antagonizes RA-induced differentiation in mESCs and F9 ECs. Furthermore, numerous reports have indicated that RA induces cell differentiation mainly through the MAPK/Erk pathway [7,16,19]. Studies have demonstrated that the suppression of Erk signaling promotes ground state pluripotency in the mouse embryos [20,21] while RA-induced differentiation of F9 cells also requires MEK1/2 activation [7]. F9 teratocarcinoma cells can be induced with retinoic acid to differentiate into parietal or visceral endoderm [22] while RA induces ECs and ESCs into several different cell types, such as primitive endoderm-like cells, neuronal cells, cardiomyocytes and pancreatic b cells in a time and concentration-dependent manner [23]. However, few reports have investigated whether microRNAs (miRNAs) are involved in the MAPK pathway that results in differentiation. Thus, the mechanism through which RA regulates cell differentiation via Erk signaling needs to be further studied and elucidated.

MicroRNAs (miRNAs) are small noncoding RNAs that consists of 21–25 nucleotides and are assembled into RNA-induced silencing complexes, which target mRNAs for silencing via the base pairing of the miRNA sequence and mRNA 3′ untranslated region (3′-UTR ). They are evolutionarily conserved and play important roles in differentiation [24,25]. For example, mir-134 modulates the differentiation of mouse ESCs by attenuating Nanog and LRH1 [26,27] while mir-200a/b/c, which is down-regulated by RA, can improve the pluripotent genes Nanog and Oct4 (6). Furthermore, RA can reduce miR-290-295 while miR-290-295 can repress Pax6 to antagonize embryonic stem cell differentiation [28]. It also showed that RA-induced up-regulation of miR-219 can promote the differentiation of embryonic stem cells into neural cells [29]. It has been reported that PD can maintain mESC pluripotency by regulating miRNAs [16,30] but little is known about the miRNA that is regulated by RA and is involved in the MAPK/Erk pathway during the process of pluripotent cell differentiation.

MiR-485 is known to be involved in cancer progression. It can suppress breast cancer cells [31] and targets PGC-1α in liver cancer [32]. α/β-hydrolase domain-containing 2 (Abhd2), which is a member of the alpha/beta hydrolase family, is also involved in cancer [33] but few reports have implicated either of these molecules in cell differentiation [34]. Based on our early work, RA and PD were found to both cause significant changes in miR-485 [5,16] and thus, we speculate that miR-485 may play a role in pluripotent cell differentiation.

In this study, we discovered that miR-485 plays a role in RA-induced F9 EC cell differentiation. Our data demonstrate that Abhd2 is a downstream target of miR-485 while both are associated with the regulation of Erk1/2 phosphorylation in F9 EC cells. Additionally, PD can induce the transcription of miR-485 to elevate Abhd2 levels and RA can induce Abhd2 by improving the transcription of miR-485. These findings imply that RA promotes F9 EC cell differentiation at least partly through miR-485 via Abhd2-regulated Erk1/2 phosphorylation.

## 2. Results

### 2.1. miR-485 Is Involved in RA-Induced Differentiation through the MAPK/Erk Pathway and Abhd2 Is a Target of miR-485

**Figure 1:**
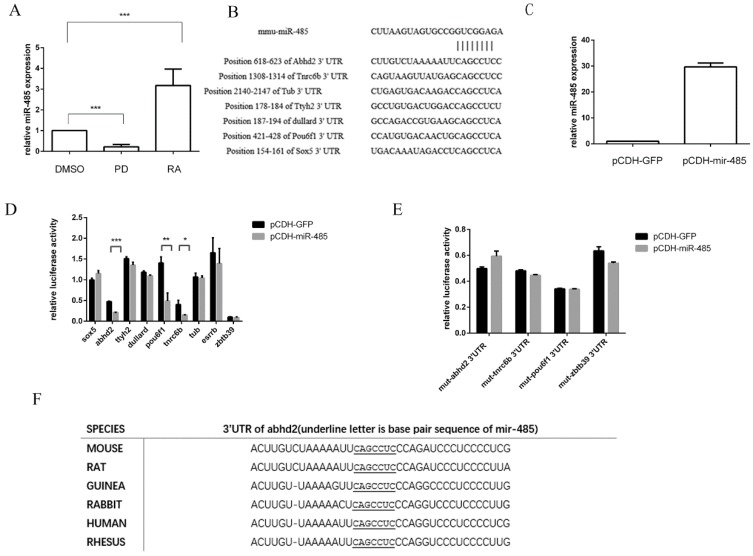
miR-485 is involved in RA-induced differentiation through the MAPK/Erk pathway and Abhd2 is a target of miR-485. (**A**) qPCR analysis of mir-485. Normally cultured F9 ECs were treated with RA (2 μM) or an equal volume of PD or DMSO for 36 h. Data are presented as mean ± SD of three independent experiments. (**B**) 3′-UTR analysis of nine target genes containing putative regions that match the seed sequence of miR-485. (**C**) qPCR analysis of miR-485 expression in F9 EC cells transfected with vector pCDH-GFP or pCDH-miR-485 for 36 h. (**D**) Dual-luciferase reporter assay of 3′-UTR of nine genes. psiCHECK2 Plasmids containing 3′-UTR of nine genes was transfected into F9 ECs, after 36 h, the luciferase activity was determined. (**E**) Dual-luciferase reporter assay of mutant 3′-UTR of nine genes. Data are presented as the mean ± SD of three independent experiments (* *p* < 0.05; ** *p* < 0.01; *** *p* < 0.001). (**F**) Sequence alignment of mouse (Mus) miR-485 with the 3′-UTRs of Abhd2 of mouse, rat, guinea, rabbit, human, rhesus.

To identify the miRNAs that are involved in the MAPK/Erk pathway, which is involved in differentiation, the small RNA sequences were analyzed from J1 mESCs treated for 24 h with 1 μM PD or from control cells treated with an equal volume of DMSO. This showed that miR-485 is significantly down-regulated by PD [16] and we identified whether miR-485 can be suppressed by PD in F9 ECs. The same effect was found in F9 ECs (Figure 1A), indicating that miR-485 may play a role in the MAPK/Erk pathway. To determine whether the MAPK/Erk pathway is involved in the differentiation of pluripotent cells, we examined whether the expression of miR-485 was altered during RA-induced differentiation. A total of 2 μM RA or an equal volume of DMSO was used to determine whether miR-485 might be involved in the differentiation of F9 ECs and miR-485 increased after RA treatment at the transcriptional level (Figure 1A). As a result, we decided to focus on miR-485 in this study since it may be involved in RA-induced differentiation via the MAPK/Erk pathway.

To identify the target genes of miR-485, the miRNA target-prediction programs, namely miRbase, miRanda, PicTar and TargetScan, were used. Using these different algorithms, nine genes were selected as possible miR-485 targets and their 3′-UTRs containing the complementary base pairs to the seed sequence of miR-485 were cloned for further study (Figure 1B). We verified that the transcriptional level of miR-485 increased by the transfection of vector pCDH-miR-485 in F9 ECs (Figure 1C). After this, we used a luciferase reporter under the control of pCDH-miR-485 (pCDH-GFP) to examine the nine putative targets in HEK293T cells. The overexpression of miR-485 leads to the down-regulation of luciferase activity in cells containing 3′-UTR of Abhd2, pou6f1 and tnrc6b luciferase reporter vectors (Figure 1D). It is important to note that miR-485 had no effect when the seed sequences of these 3′-UTRs were mutated (Figure 1E). This showed that the activity of luciferase was repressed by miR-485 through the seed sequence of their 3′-UTRs. Furthermore, miR-485 had the most significant effect on the 3′-UTR of Abhd2 and this potential binding site for miR-485 within the 3′-UTR is highly conserved between species (Figure 1F). These data demonstrated that miR-485 targets the 3′-UTR of Abhd2 through its binding site within the 3′-UTR of Abhd2.

### 2.2. Abhd2 Is a Target of miR-485 and Both Molecules Are Regulated by RA and PD

**Figure 2:**
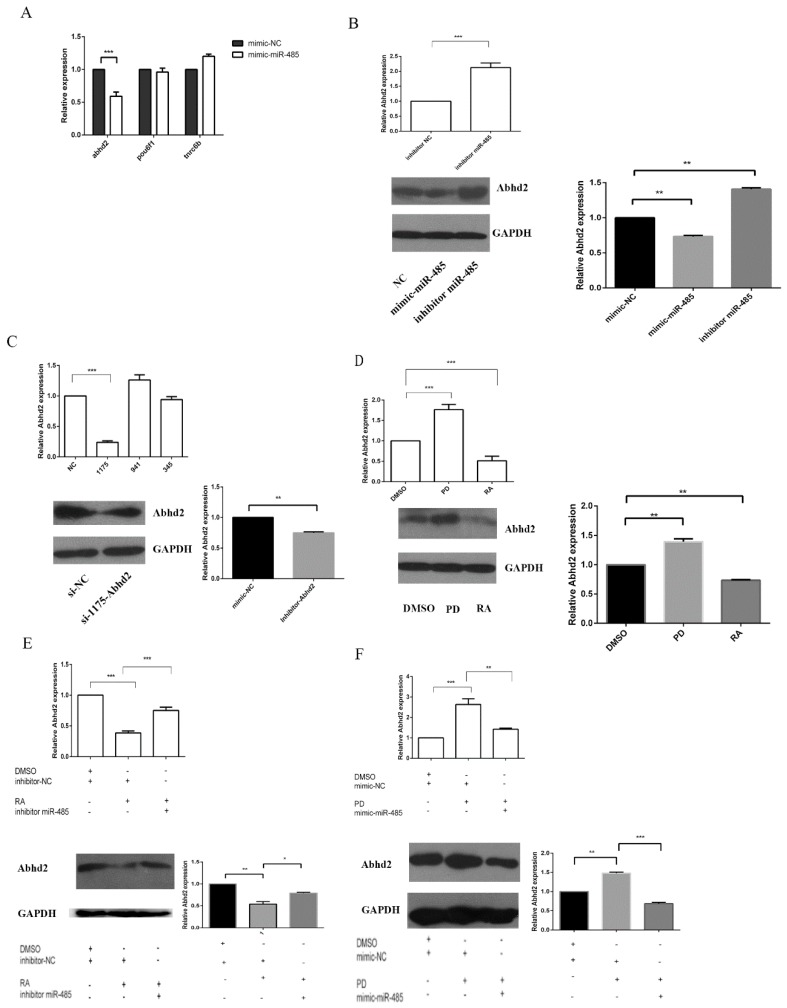
RA, PD and mir-485 influence Abhd2 at both transcriptional level and post-transcriptional level. (**A**) RT-qPCR analysis for the representative genes that may be regulated by miR-485. 36 h after being transfected with mimic NC or mimic-miR-485, F9 ECs were gathered. The relative expression of three genes were determined by qPCR analysis. Gapdh normalized qPCR data were used to show the expression change of the indicated genes. Data are presented as the mean ± SD of three independent experiments (* *p* < 0.05; ** *p* < 0.01; *** *p* < 0.001). (**B**) Up panel: qPCR analysis of Abhd2. Bottom panel: Western Blot analysis of Abhd2. F9 ECs were transfected with miR-485 or inhibitor of miR-485, after 36 h, the Abhd2 expression was determined. Gapdh normalized qPCR and Western Blot data. (**C**) Up panel: qPCR analysis of the effect of the inhibitor of Abhd2. Bottom panel: Western Blot analysis of the effect of the inhibitor of Abhd2. Three kinds of si-Abhd2 were transfected into F9 ECs for 36 h respectively. the expression of Abhd2 were determined by qPCR and Western Blot analysis. Gapdh was used to normalize template levels. The number 1175,941,345 is the code name of three kinds of siRNA of Abhd2 (**D**) Up panel: qPCR analysis of Abhd2 expression in F9 EC cells treated by DMSO, RA or PD for 36 h. Bottom panel: Western Blot analysis of Abhd2 expression in F9 EC cells treated by DMSO, RA or PD for 36 h. (**E**) Up panel: qPCR analysis of Abhd2 expression in F9 EC cells transfected with NC or inhibitor of miR-485. Bottom panel: Western Blot analysis of Abhd2 expression in F9 EC cells transfected with NC or inhibitor of miR-485. After 6h transfection, F9 ECs with the inhibitor of miR-485 or negative control were culture in DMSO or RA contain medium for an additional 30 h. Gapdh was used to normalize template levels. (**F**) Up panel: qPCR analysis of Abhd2 expression in F9 EC cells transfected with NC or mimic of miR-485. Bottom panel: Western Blot analysis of Abhd2 expression in F9 EC cells transfected with NC or mimic of miR-485. After 6h transfection, F9 ECs with the mimic of miR-485 or negative control were culture in DMSO or PD contain medium for an additional 30 h. Gapdh was used to normalize template levels. and for Western Blot, Gapdh was used to normalize template levels. The inhibitor NC is same as mimic NC.

To determine whether the targets of miR-485 were down-regulated by miR-485 at the transcriptional and post-transcriptional levels, mimics and antisense oligonucleotides of miR-485 were transfected into F9 ECs. These samples were subsequently analyzed by immunoblotting and reverse transcription quantitative real-time polymerase chain reaction (RT-qPCR). As shown in Figure 2A, Abhd2, but not pou6f1 and tnrc6b, was suppressed by the mimic of miR-485. In contrast, Abhd2 was up-regulated by the antisense oligonucleotides of miR-485 at the transcriptional level. Consistently, Abhd2 exhibited the same responses at the post-transcriptional and the transcriptional levels (Figure 2B). siRNAs that targeted Abhd2 and the negative control (si-NC) were transfected into F9 ECs to determine their knockdown efficiency and siRNA-1175 (si-Abhd2) was the most efficient in reducing Abhd2 expression (Figure 2C).

To confirm that miR-485 and Abhd2 is involved in the differentiation process of F9 ECs, the small molecules of RA and PD were used to induce the cell processes. F9 ECs were treated with DMSO, RA or PD. After 36 h, the total protein or RNA was collected for immunoblotting and RT-qPCR, respectively. As expected, Abhd2 was up-regulated by PD and down-regulated by RA (Figure 2D). Those results indicated that Abhd2 was regulated by RA and PD and it may be involved in the MAPK/Erk pathway. To obtain further verification of the involvement of miR-485 in the process by which RA or PD regulates Abhd2, we treated F9 ECs with RA simultaneously. After this, the cells were transfected with the antisense oligonucleotides of miR-485 (inhibitor of miR-485). After 36 h, the total protein was collected for immunoblotting and RT-qPCR. Figure 2E shows that Abhd2 was promoted by the antisense oligonucleotides of miR-485 after RA treatment. This indicated that Abhd2 reduction could be suppressed by the antisense oligonucleotides of miR-485 while RA existed. Furthermore, Figure 2F shows that Abhd2 could be suppressed by the mimic of miR-485 after PD treatment. Abhd2 reduction could be improved by the mimic of miR-485 while PD existed. These results indicate that RA and PD regulate Abhd2 via miR-485 in F9 ECs.

### 2.3. miR-485 and Abhd2 Are Involved in the MAPK/Erk Pathway during RA-Induced Differentiation

**Figure 3:**
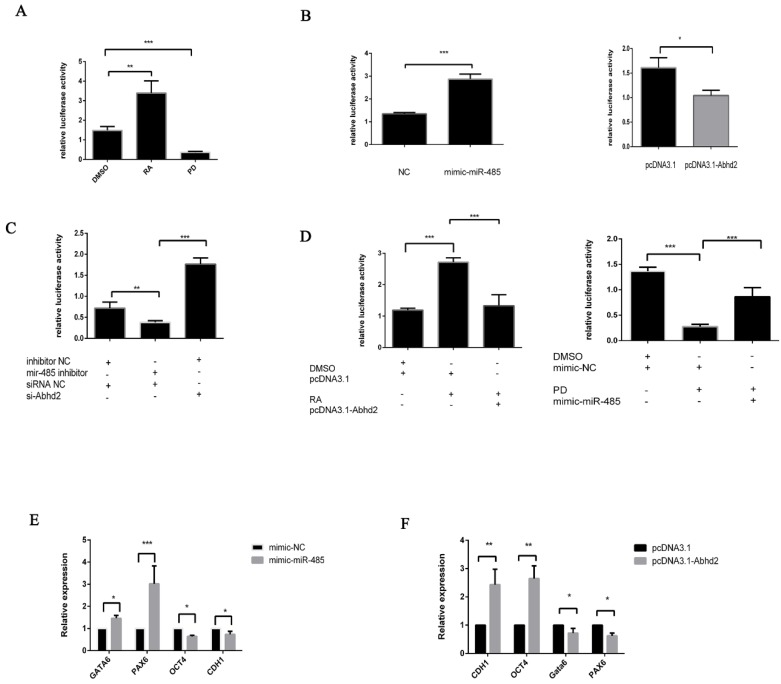
miR-485 and Abhd2 are involved in the MAPK/Erk pathway during RA-induced differentiation. (**A**) dual-luciferase reporter assay of MAPK/ERK pathway influenced by RA and PD. Pathway reporter luciferase vector pSRE-TA-luc (including negative control) and internal control pRL-SV40 were co-transfected into F9 ECs for 36 h. after transfection 6 h, 2 μM RA, PD or DMSO was added into medium. Luciferase activity is presented relative to negative control pTA-luc. Data are presented as the mean ± SD of three independent experiments (* *p* < 0.05; ** *p* < 0.01; *** *p* < 0.001). (**B**) Left panel: dual-luciferase reporter assay of MAPK/ERK pathway influenced by miR-485. Right panel: dual-luciferase reporter assay of MAPK/ERK pathway influenced by Abhd2. Pathway reporter luciferase vector pSRE-TA-luc pRL-SV40 were co-transfected with NC, mimic-485, pcDNA3.1, pCDNA3.1-Abhd2. (**C**) dual-luciferase reporter assay of MAPK/ERK pathway influenced by suppressing miR-485 and Abhd2. pRL-SV40 were co-transfected with inhibitor mir-485, si-NC or si-Abhd2 into F9 ECs for 36 h. Luciferase activity is presented relative to negative control pTA-luc. (**D**) Left panel: dual-luciferase reporter assay of MAPK/ERK pathway influenced by RA and Abhd2. Pathway reporter luciferase vector pSRE-TA-luc and internal control pRL-SV40 were co-transfected with pCDNA3.1, pCDNA3.1-Abhd2 into F9 ECs for 36 h. after transfection 6 h, 2 μM RA or DMSO was added into medium. Luciferase activity is presented relative to negative control pTA-luc. Right panel: dual-luciferase reporter assay of MAPK/ERK pathway influenced by PD and miR-485. Pathway reporter luciferase vector pSRE-TA-luc and internal control pRL-SV40 were co-transfected with mimic-NC or mimic-miR-485 into F9 ECs for 36 h. after transfection 6 h, 2 μM PD or DMSO was added into medium. Luciferase activity is presented relative to negative control pTA-luc. (**E**) qPCR analysis of downstream genes in MAPK/ERK pathway in F9 EC cells transfected with NC or mimic of miR-485 for 36 h. Gapdh was used to normalize template levels. (**F**) qPCR analysis of downstream genes in MAPK/ERK pathway in F9 EC cells transfected with pcDNA-3.1 or pcDNA-3.1-Abhd2 for 36 h. Gapdh was used to normalize template levels.

To confirm that miR-485 is involved in the MAPK/Erk pathway, we first tested whether RA and PD could influence the signaling pathway using the firefly luciferase vector pSRE-TA-luc, which is the signaling pathway vector of MAPK/Erk pathway. F9 ECs were treated with DMSO, RA or PD for 36 h before being transfected with pSRE-TA-luc. This showed that RA stimulated the MAPK/Erk pathway whereas PD antagonized it (Figure 3A). Similar results have been obtained with mESCs [5]. After this, pSRE-TA-luc was cotransfected into F9 ECs with pCDH-miR485 for 36 h and a dual luciferase assay was performed to determine if miR-485 was involved in the MAPK/Erk pathway. This showed that the MAPK/Erk pathway could also be suppressed by miR-485 (Figure 3B). Similar experiments on the target of miR-485, Abhd2, were also performed and revealed that the activity of the MAPK/Erk pathway could be enhanced by Abhd2 (Figure 3B). To verify that these effects were due to miR-485 and Abhd2, the siRNA of miR-485 and Abhd2 were also transfected into F9 ECs for 36 h. This showed that the MAPK/Erk pathway was suppressed by the Abhd2 inhibitor, whereas it was enhanced by the antisense oligonucleotides of miR-485 (Figure 3C). To verify that the influence of RA and PD on the MAPK/Erk pathway occurs via miR-485 and Abhd2, F9 ECs were treated for 36 h with either 2 μM RA with Abhd2 or 2 μM PD with miR-485. The activity of the MAPK/Erk pathway was reduced after RA treatment compared to the RA treatment with transfection of Abhd2 (Figure 3D). Once again, the activity MAPK/Erk pathway increased after PD treatment compared to the PD treatment with transfection of miR-485. Furthermore, the activity of the MAPK/Erk pathway was improved after PD treatment compared to the PD treatment with transfection of Abhd2 (Figure 3E). To further prove that miR-485 is involved in MAPK/Erk pathway, miR-485 was transfected into F9 ECs for 36 h before the downstream genes in MAPK/Erk pathway were detected (Figure 3F). The pluripotent marker Oct4 and CDH1 were down-regulated by miR-485 while the endoderm and differential markers GATA6, Pax6 were up-regulated by miR-485. Oct4 and CDH1 was promoted by Abhd2, and GATA6 as well as Pax6 were suppressed by Abhd2. These results indicate that RA and PD affect the MAPK/Erk pathway at least partially via miR-485 and Abhd2.

### 2.4. RA Regulates Erk1/2 Phosphorylation through miR-485 and Abhd2 in F9 ECs

**Figure 4:**
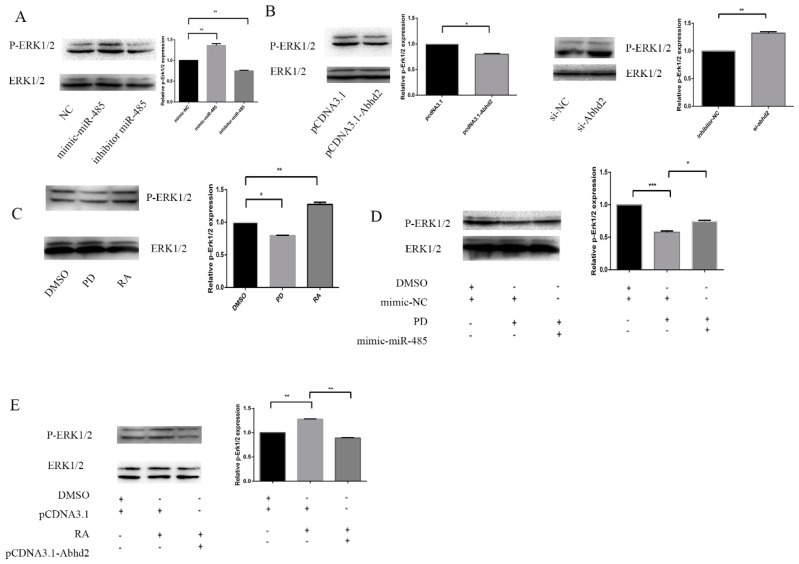
RA regulates Erk1/2 phosphorylation through miR-485 and Abhd2 in F9 ECs (**A**) Western Blot analysis with statistical analysis of phosphorylation of ERK1/2 that were modulated by miR-485. F9 ECs were transfected with mimic-NC, mimic-miR-485 and inhibitor of miR-485 for 36 h. phosphorylation of ERK1/2 level was analyzed by Western Blot. Total ERK1/2 was used to normalize template levels. Data are presented as the mean ± SD of three independent experiments (* *p* < 0.05; ** *p* < 0.01; *** *p* < 0.001). (**B**) Western Blot analysis with statistical analysis of phosphorylation of ERK1/2 that were modulated by Abhd2. F9 ECs were transfected with pCDNA3.1, pCDNA3.1-Abhd2, si-NC and si-Abhd2 for 36 h, Total ERK1/2 was used to normalize template levels. (**C**) Western Blot analysis with statistical analysis of phosphorylation of ERK1/2 that were altered by RA or PD. F9 ECs were treated with 2 μM PD, RA or DMSO respectively for 36 h. Total ERK1/2 was used to normalize template levels. (**D**,**E**) Western Blot analysis with statistical analysis of phosphorylation of ERK1/2 with different treatment. (**D**) F9 ECs were transfected with mimic-NC or mimic-miR-485, 6 h after transfection, the cells were treated with 2 μM PD or DMSO. phosphorylation of ERK1/2 level was analyzed by Western Blot. Total ERK1/2 was used to normalize template levels. (**E**) F9 ECs were transfected with pCDNA3.1, pCDNA3.1-Abhd2, 6 h after transfection, the cells were treated with 2 μM RA or DMSO. Total ERK1/2 was used to normalize template levels.

A previous study showed that RA can modulate the phosphorylation of Erk1/2, which subsequently accelerates the cell differentiation. Furthermore, they found that the phosphorylation of Erk1/2 can be up-regulated by inhibiting Abhd2 in cancer cells [35,36]. Therefore, we wanted to determine whether the phosphorylation of Erk1/2 can be regulated by miR-485 in F9 ECs. As shown in Figure 4A, Erk1/2 phosphorylation was up-regulated by a miR-485 mimic and down-regulated by the antisense oligonucleotides to miR-485. To determine whether Erk1/2 phosphorylation is altered by miR-485 through a reduction in Abhd2, we first confirmed that siRNA-1175 can play the same role in F9 ECs. The results showed that the phosphorylation of Erk1/2 can be down-regulated by pCDNA3.1-Abhd2 in F9 ECs (Figure 4B). We also repeated the experiment, which showed that the phosphorylation level of Erk1/2 can increase after RA treatment or reduce after PD (Figure 4C). To provide further confirmation, we transfected Abhd2 into F9 ECs after RA treatment and transfected mimic-mir-485 with PD treatment in order to identify whether RA functions via miR-485 and Abhd2. As shown in Figure 4D, the reduction in Erk1/2 phosphorylation by PD was suppressed by miR-485. Furthermore, RA-induced phosphorylation of Erk1/2 was blocked by Abhd2 (Figure 4E). Together, all these results indicate that RA regulates Erk1/2 phosphorylation via miR-485 and Abhd2 in F9 ECs.

### 2.5. RA Induces Cell Differentiation through Induceing Nanog by MiR-485

**Figure 5:**
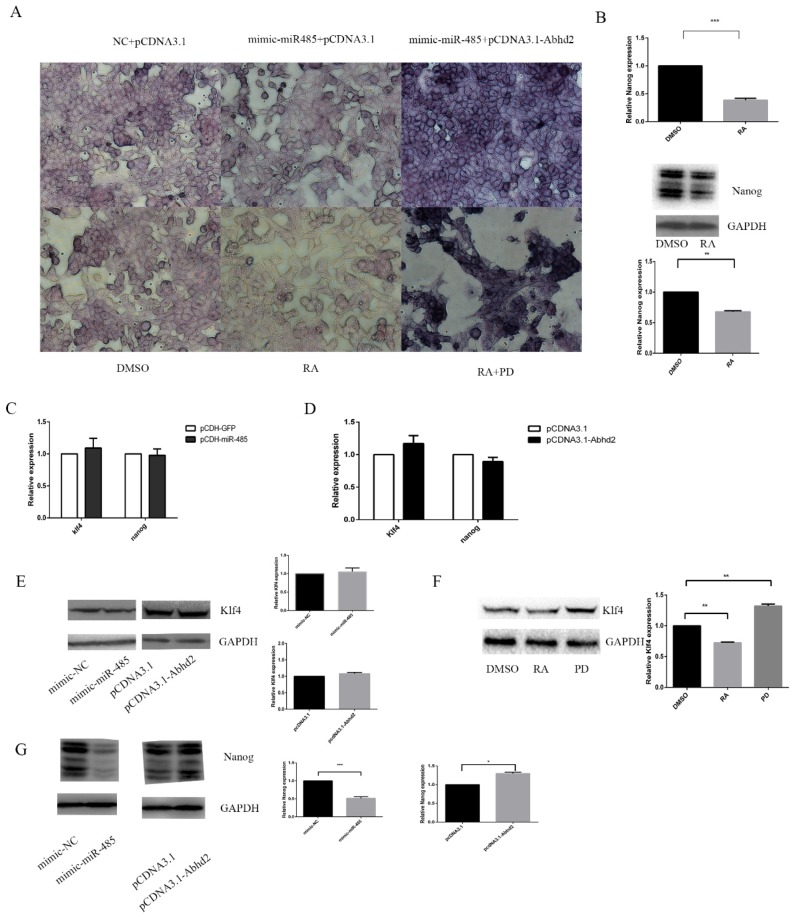
miR-485 and abhd2 influence cell differentiation by modulating Nanog. (**A**) Alkaline phosphatase activity staining of F9 ECs with different treatments. The vector pCDNA3.1 and pCDNA3.1-Abhd2, mimic-NC or mimic-miR-485 were transfected into F9 ECs for 36 h. after transfection 6 h, 2 μM RA, PD or DMSO was added into medium. The activities of AP were shown, and cells were captured under 400×. (**B**) qPCR and Western Blot analysis of Nanog. Up panel: qPCR analysis of nanog after 36 h of treatment with DMSO or RA. Bottom panel: Western Blot analysis of Nanog after 36 h of treatment with DMSO or RA, and protein bands were quantified by using ImageJ software and calculated by using the samples normalized to Gapdh. Data are presented as the mean ± SD of three independent experiments (* *p* < 0.05; ** *p* < 0.01; *** *p* < 0.001). (**C**) qPCR analysis of the expression of Klf4 and Nanog after 36 h transfection with mimic-NC or mimic-miR-485. (**D**) qPCR analysis of the expression of Klf4 and Nanog after 36 h transfection with pCDNA3.1 or pCDNA3.1-Abhd2. (**E**) Western Blot analysis of the expression of Klf4. F9 ECs were cultured in DMEM medium were transfected with pCDNA3.1, pCDNA3.1-Abhd2, mimic-NC or mimic-miR-485 for 36 h. Left panel: Western Blot analysis of the expression of Klf4. Right panel: the statistical analysis of Western Blot. (**F**) Western Blot analysis of Klf4 after 36 h of treatment with DMSO, RA or PD. Left panel: Western Blot analysis of the expression of Klf4. Right panel: the statistical analysis of Western Blot. (**G**) Western Blot analysis of the expression of Nanog after 36 h transfection with mimic-NC or mimic-miR-485, pCDNA3.1 or pCDNA3.1-Abhd2. Left panel: Western Blot analysis of the expression of Nanog. Right panel: the statistical analysis of Western Blot.

Undifferentiated pluripotent cells exhibit higher levels of alkaline phosphatase (AP) activity and thus, AP staining was used to test cellular pluripotency. The AP activity of F9 ECs was lower after RA compared to a DMSO control. Furthermore, this activity improved after PD, indicating that the pluripotency of F9 ECs was blocked by RA while it was promoted by PD in the presence of RA (Figure 5A). Similarly, the pluripotency of F9 ECs was reduced by miR-485 but this effect was reduced by the addition of Abhd2 (Figure 5A).

Nanog and Klf4 is core pluripotent markers of ESC and ECs, which is involved in the MAPK/Erk pathway [14,37]. Previous studies have shown that RA leads to cell differentiation with the down-regulation of Nanog at both transcriptional and post-transcriptional levels in ESCs and we obtained the same results in F9 ECs (Figure 5B). However, miR-485 and Abhd2 had no effect on klf4 or Nanog at the transcriptional level (Figure 5C,D), but KLF4 can be regulated by RA or PD at the post-transcriptional level (Figure 5F). After this, we examined the post-transcriptional levels of Nanog. As shown in Figure 5G, Nanog could be suppressed by miR-485 and improved by Abhd2 at the post-transcriptional level. However, Klf4 remained unaltered (Figure 5E). Together, these results reveal that RA treatment leads to cell differentiation via miR-485, with this process involving Erk1/2 phosphorylation.

## 3. Discussion

RA treatment can lead to pluripotent cell differentiation and this process involves a variety of genes [29,38,39]. For example, c-Fos decreased during the RA-induced F9 cell differentiation, which regulated the phosphorylation of ETS transcription factor ELK1 [7]. GATA6, FGF4 and PAX6, which are downstream genes of the MAPK/Erk pathway, contribute to the RA-induced F9 cell differentiation [17,28,40]. Previous studies have shown that RA-dependent cell differentiation is partly caused by the activation of the MAPK pathway and it has been shown that mesenchymal stem cells (MSCs) and ESCs both can differentiate after the activation of the MAPK/Erk pathway [21,41,42]. RA drives F9 EC differentiation along the somatic lineage and this function is mediated by the activation of the nuclear RA receptors, RARα, β and γ. All these receptors can become phosphorylated through the MAPK/Erk signaling pathway [36,43]. The activation of the MAPK pathway is involved in the differentiation of pluripotent cells and for this reason, the small-molecule PD, an inhibitor of the MAPK pathway, can maintain the pluripotency of stem cells [44].

RA induces numerous alterations in microRNAs. For example, miR-10a targets histone deacetylase 4 to influence the NF-kB pathway during smooth muscle cell (SMC) differentiation from embryonic stem cells [45]. Furthermore, miR-134 can target Nanog and LRH1 directly to cause the differentiation of ESCs [26]. However, only a few reports have focused on the MAPK/Erk signaling pathway.

miR-485 is involved in the suppression of tumors by inhibiting cell growth [31] and RA can inhibit cell growth by suppressing P2X7R during the differentiation of ESCs [46]. Our results showed that miR-485 was up-regulated by RA during the differentiation of F9 ECs and it can be suppressed by PD, which showed that miR-485 may play a role in the MAPK/Erk signaling pathway. Nine targeted genes were identified to test the role of miR-485 in this process. It was expected that the genes that maintain their pluripotency may be the targets of miR-485. However, the estrogen related receptor, beta (Esrrb), which is a direct Nanog Target gene for pluripotency maintenance [47], is not significantly suppressed by miR-485. However, this may be due to miR-485 being obstructed by the mRNA structure of Esrrb. However, the response gene of miR-485, Abhd2, was reported to be able to suppress the phosphorylation of Erk1/2 in ovarian cancer [35] and our study showed that Abhd2 had the same effect during the differentiation of F9 ECs. Besides, in Figure 3D, the luciferase activity in the cells treated with DMSO was similar to that treated with retinoic acid and pcDNA3.1-Abhd2 vector, it may be depend on the expression of Abhd2. Therefore, we verified the expression of Abhd2, and the result showed that the expression of Abhd2 is similar in two treatment (Supplemental Figure S1), indicating that the MAPK/Erk signaling pathway is regulated by the amount of Abhd2.Above all, miR-485 may target Abhd2 to participate in the process of differentiation of F9 ECs. Furthermore, this indicated that miR-485 may be involved in the MAPK/Erk pathway during RA-induced differentiation. Our results also showed that miR-485 could influence the downstream genes in the MAPK/Erk pathway.

**Figure 6:**
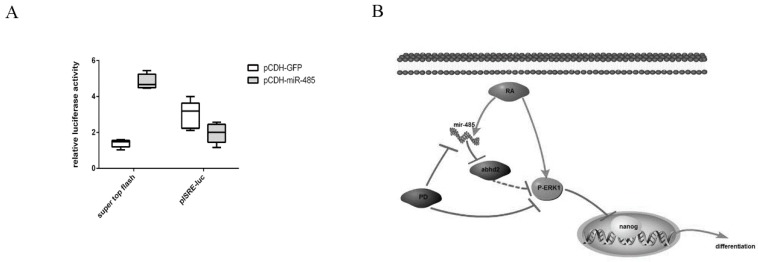
(**A**) miR-485 is involved in Wnt and JAK/STAT pathway. Pathway reporter luciferase vector pISRE-luc and super top flash and internal control pRL-SV40 were co-transfected with pCDH-GFP and pCDH-miR-485 into F9 ECs for 36 h. Luciferase activity was presented relative to negative control pTA-luc. Data are presented as the mean ± SD of six independent experiments. (**B**) Schematic diagram of the RA-induced cell differentiation by ERK1/2.

Nanog occupies a central position in the pluripotency network and is a highly expressed pluripotency marker in ES and EC cells. During cellular differentiation, its level decreases rapidly [48,49] and RA can reduce the pluripotent marker Nanog in F9 ECs, which leads to cell differentiation [48]. Klf4 is a key TF regulating the gene expression program of naïve pluripotency, it mediates the basic nuclear organization at the Oct4 locus which contributes to maintaining the pluripotency of ESCs [50,51] and the stability of Klf4 and Nanog is influenced by the MAPK/Erk pathway [14,37]. Thus, we proposed that miR-485 mediates the differentiation of F9 ECs via reducing Nanog and Klf4 expression. However, Nanog and Klf4 exhibited no changes at the transcriptional level. This may be due to miR-485 acting by promoting Erk1/2 phosphorylation, which subsequently leads to a reduction in Nanog and Klf4 stability due to their ubiquitination [14]. Thus, miR-485 may regulate Nanog and Klf4 at the post-transcriptional level. The Wnt pathway is activated by miR-485 in F9 ECs (Figure 6A). Klf4 is a pluripotency marker that can be up-regulated by this Wnt pathway [12]. Thus, since miR-485 promotes both MEK/Erk and Wnt signaling, this may explain why it did not change the expression of Klf4. During the RA-induced differentiation of F9 cells, the protein expression of Klf4 was decreased mainly by the increased miR-485 expression and the activated Erk signaling (Figure 5F). However, miR-485 overexpression in F9 cells did not reduce the expression of Klf4 obviously (Figure 5E), because it also activated the activity of Wnt signaling (Figure 6A), which may promote the expression of Klf4. Except for Erk and Wnt signaling, the expression of Klf4 can also be regulated by other pathways, like PI3K/Akt [52], in RA-induced F9 cells. Thus, the relative low level of Klf4 is the comprehensive outcome of many factors induced by RA in F9 cells. The miR-485 may partly rescue the Klf4 expression by Wnt pathway, to avoid the excessive suppression of Klf4 by other ways during the RA-induced differentiation. A certain amount of Klf4 is necessary for the repair of DNA damages during differentiation [53]. The Wnt signaling cannot only maintain pluripotency, but also antagonize apoptosis [54,55]. Our results also show that the JAK/STAT pathway is repressed. As stat3 is a target of miR-485 in hepatocellular carcinoma cells (human), this may have the same effect in mice [56].

We have shown that Abhd2 acts as a target of miR-485 in the MAPK pathway and that it regulates the phosphorylation of Erk1/2 in F9 ECs. Furthermore, Abhd2 is a factor that promotes the maintenance of pluripotency. Collectively, our results provide a new insight into the function of miRNAs in the MAPK pathway during RA-induced cell differentiation.

Our results indicated that miR-485 participates in several mechanisms of differentiation. However, there needs to be further studies on how miR-485 and Abhd2 regulate the phosphorylation of Erk1/2 and in the future, the mechanisms through which Abhd2 influences differentiation also need to be clarified in order for RA-induced cell differentiation to be fully understood.

## 4. Materials and Methods

### 4.1. Cell and Mycobacterial Culture, and Infection

HEK293T cells and F9 teratoma cells were obtained from the American Type Culture Collection (ATCC, Manassas, VA, USA) and cultured in Dulbecco’s modified Eagle’s medium (DMEM) in the sterile cell wells which were purchased from Nunclon (Roskilde, Denmark). The cells were supplemented with 10% (*v*/*v*) FBS. All cells were maintained at 37 °C and 5% CO_2_ in a humidified incubator.

Transfections were performed by using Lipofectamine 2000 (Life Technologies Inc., Carlsbad, CA, USA) according to the manufacturer’s instructions. All cell culture reagents were purchased from Gibco (Invitrogen, Carlsbad, CA, USA) unless indicated.

### 4.2. PD/RA Treatment

PD (Santa Cruz, CA, USA) was dissolved in dimethyl sulfoxide (DMSO) and added to cell medium at a final concentration of 2 μM. RA (Santa Cruz, CA, USA) was dissolved in DMSO at a final concentration of 2 μM. An equal volume of DMSO added in another well was used as a control.

### 4.3. Reverse Transcription PCR and Quantitative Real-Time PCR Analysis

Total RNA was extracted from treated cells using Trizol Reagent (Life Technologies) according to the manufacturer’s protocol. Total RNA (1 μg) was reverse-transcribed using a PrimeScript RT reagents kit (TaKaRa, Dalian, China) with the protocol: Reactions were carried out at 37 °C for 15 min and 85 °C for 5 s. The amount of RNA was determined by real-time PCR with an ABI StepOne Plus PCR system (Applied Biosystems, Foster City, CA, USA) using SYBR Premix Ex Taq II (TaKaRa). The following conditions were used for qPCR: 30 s at 95 °C, and 40 cycles of 5 s at 95 °C and 30 s at 60 °C, and the melting curve was analyzed to ensure that a single PCR product was obtained. Data were collected after each annealing step. Glyceraldehyde-3-phosphate dehydrogenase (Gapdh) was used as an endogenous control to normalize for the differences in the amount of total RNA in each sample. All primer sequences used for qPCR examination were obtained from the PrimerBank (http://pga.mgh.harvard.edu/primerbank/) and shown in the Supplementary Table (Table S1)

For miRNA RT-qPCR analysis, the total RNA (2 μg) was reverse-transcribed using a miScript II RT kit (Qiagen, Shanghai, China) to obtain mature miRNA. Real-time PCR was performed in triplicate for each sample by using miScript SYBR Green PCR kit (Qiagen). The endogenous reference RNA RNU6B was used to normalize the amount of template added for each sample. The following primers were used: q-miR485-5p: GGAGAGGCTGGCCGTGA and Universal miRNA Forward: TGAATCGAGCACCAGTTACGCATGCCGAGGTCGACTTCCTAGA.

### 4.4. Plasmid Constructs

CDS sequence of Abhd2 was amplified from the genomic DNA of ES cells by PCR. The PCR primers were as follows: forward primer: 5′-CGGAATTCGCCACCATGGACTACAAAGACGATGACGACAAGATGAATGCCATGCTAGAGACCC-3′ (underlined letters indicate an *EcoR*I restriction site) and reverse primer: 5′-ATAAGAATGCGGCCGCTCATTCCAACTCGGCCTCCATCTGCTCC-3′ (underlined letters indicate a *Not*I restriction site). The PCR sample was ligated into the pcDNA 3.1. a fragment carrying pre-miR-485 was amplified from genomic DNA of J1 ESCs and cloned into the pCDH-CMV-MCS-EF1-coGFP vector (System Biosciences, Mountain View, CA, USA). The PCR primers were as follows: forward primer: 5′-GCTCTAGACTACCACAGGAGCTTCCAGAATA-3′ (underlined letters indicate a *Xba*I restriction site) and reverse primer: 5′-ATAAGAATGCGGCCGCTAGCTTGGACACTGGGATAACTG-3′ (underlined letters indicate a *Not*I restriction site).

The 3′-UTR of genes that contain putative miRNA binding sites were amplified from J1 ESC cDNA through PCR and then cloned into psiCHECK-2 Vector (Promega) for Luciferase assays. All primer sequences used were shown in the Supplementary Table (Table S2)

### 4.5. RNA Interference

Short interfering RNAs (siRNAs) that target mouse Abhd2 and negative control siRNA-NC were purchased from Shanghai GenePharma (Shanghai, China). siRNA sequences were shown in the Supplementary Table (Table S3). For RNA interference experiments, F9 ECs were transfected with the indicated siRNAs (50 nM final concentrations) using Lipofectamine 2000.

### 4.6. Luciferase Reporter Assays

Luciferase assays were performed by using a Dual-Luciferase Reporter Assay System (Promega, Madison, WI, USA). The luciferase reporter and pCDH-miR-485 were cotransfected into HEK293T cells using Lipofectamine 2000. After 36 h, transfected cells were lysed in passive lysis buffer for 15 min with shaking by Rockers. Firefly luciferase activity in cell lysates was measured on a VICTOR X5 Multilabel Plate Reader (PerkinElmer, Cetus, Norwalk, CA, USA) and normalized to Renilla luciferase.

Pathway reporter vector pSRE-TA-luc was purchased from Beyotime. The negative control vectors pTA-luc and the JAK/STAT pathway reporter vector pISRE-TA-luc were obtained from Clonetech (Mountain View, CA, USA). The Wnt signaling pathway reporter vector pSuperTOPFlash luciferase reporter was constructed by inserting seven copies of the TCF/LEF binding site (AGATCAAAGG) into the pTA-luc vector, The pRL-SV40 (Promega) was used to normalized to Renilla luciferase. they were cotransfected with vectors like pcDNA3.1-Abhd2 or mimic of miR-485-5p into F9 ECs by using Lipofectamine 2000 for 36 h, Luciferase activity was detected as above.

### 4.7. Western Blot Analysis

Cells were lysed with RIPA buffer (Pierce, Rockford, IL, USA) The BCA Protein Assay Reagent (Pierce) was used to quantify the protein concentration of lysates. Equal amounts of protein were resolved by 8–12% sodium dodecyl sulfate-polyacrylamide gel electrophoresis. Then the proteins were transferred to the polyvinylidene fluoride membranes (Millipore, Bedford, MA, USA). After blocking for 4 h at room temperature in 10% nonfat dry milk in TBST containing 0.05% Tween-20, the membranes were incubated with primary antibodies overnight at 4 °C and then after washes using TBST, it was incubated with HRP-conjugated secondary antibodies for 2 h at room temperature.

Primary antibodies included rabbit anti-Klf4 (Abcam, Cambridge, UK, 1:1000) Rabbit monoclonal anti-GAPDH (Sigma, 1:5000) rabbit anti-Nanog (CST, Danvers, MA, USA), Anti-ERK1/2 rabbit polyclonal antibody (sangon biotech, Shanghai, China, 1:1000), Anti-ERK1/2(Thr202/Tyr204)) rabbit polyclonal antibody (sangon biotech, 1:1000), Rabbit Polyclonal anti-ABHD2 (Proteintech, Rosemont, IL, USA, 1:1000) and anti-rabbit/mouse horseradish peroxidase-conjugated secondary antibody were obtained from the Beyotime Institute of Biotechnology (Jiangsu, China ).

### 4.8. Statistical Analysis

The data in graphs are expressed as the mean ± SD. The difference between two groups was compared by a two-tailed paired Student’s *t*-test, and significance was set at *p*-values < 0.05. The difference between three or more groups was compared by an analysis of variance (ANOVA), and significance of differences was determined by post hoc testing. Multiple comparisons between the groups were performed using the S-N-K method.

## 5. Conclusions

We explored the mechanisms by which RA induces cell differentiation via miRNA. Our results suggest that RA-induced miR-485 is involved in the F9 EC differentiation and that miR-485 plays its role by targeting Abhd2. This process modulates Erk1/2 phosphorylation and ultimately Nanog activity. Ultimately, RA regulates miR-485 to induce embryonal carcinoma cell differentiation through the MAPK/Erk pathway (Figure 6B).

## Supplementary Materials

Supplementary materials can be found at https://www.mdpi.com/1422-0067/20/9/2071/s1.

## Abbreviations

RA	Retinoic acid
ECs	Embryonal carcinoma cells
ESCs	Embryonic stem cells
MAPK	Mitogen-activated protein kinase
Erk	Extracellular signal-regulated kinase
NC	Negative control

## References

[1] Ivanova N., Dobrin R., Lu R., Kotenko I., Levorse J., DeCoste C., Schafer X., Lun Y., Lemischka I.R. (2006). Dissecting self-renewal in stem cells with RNA interference. Nature.

[2] Wu H., Wu Y., Ai Z., Yang L., Gao Y., Du J., Guo Z., Zhang Y. (2014). Vitamin C enhances Nanog expression via activation of the JAK/STAT signaling pathway. Stem Cells.

[3] Lehtonen E., Laasonen A., Tienari J. (1989). Teratocarcinoma stem cells as a model for differentiation in the mouse embryo. Int. J. Dev. Biol..

[4] Alonso A., Breuer B., Steuer B., Fischer J. (1991). The F9-EC cell line as a model for the analysis of differentiation. Int. J. Dev. Biol..

[5] Zhang J., Gao Y., Yu M., Wu H., Ai Z., Wu Y., Liu H., Du J., Guo Z., Zhang Y. (2015). Retinoic Acid Induces Embryonic Stem Cell Differentiation by Altering Both Encoding RNA microRNA Expression. PLoS ONE.

[6] Smith E.R., Smedberg J.L., Rula M.E., Xu X.X. (2004). Regulation of Ras-MAPK pathway mitogenic activity by restricting nuclear entry of activated MAPK in endoderm differentiation of embryonic carcinoma and stem cells. J. Cell Biol..

[7] Smith E.R., Smedberg J.L., Rula M.E., Hamilton T.C., Xu X.X. (2001). Disassociation of MAPK activation and c-Fos expression in F9 embryonic carcinoma cells following retinoic acid-induced endoderm differentiation. J. Biol. Chem..

[8] Huang F.J., Lan K.C., Kang H.Y., Lin P.Y., Chan W.H., Hsu Y.C., Liu Y.C., Huang K.E. (2013). Retinoic acid influences the embryoid body formation in mouse embryonic stem cells by induction of caspase and p38 MAPK/JNK-mediated apoptosis. Environ. Toxicol..

[9] Wei Z.Z., Yu S.P., Lee J.H., Chen D., Taylor T.M., Deveau T.C., Yu A.C., Wei L. (2014). Regulatory role of the JNK-STAT1/3 signaling in neuronal differentiation of cultured mouse embryonic stem cells. Cell. Mol. Neurobiol..

[10] Mauney J.R., Ramachandran A., Yu R.N., Daley G.Q., Adam R.M., Estrada C.R. (2010). All-trans retinoic acid directs urothelial specification of murine embryonic stem cells via GATA4/6 signaling mechanisms. PLoS ONE.

[11] Du J., Wu Y., Ai Z., Shi X., Chen L., Guo Z. (2014). Mechanism of SB431542 in inhibiting mouse embryonic stem cell differentiation. Cell. Signal..

[12] Ai Z., Shao J., Wu Y., Yu M., Du J., Shi X., Shi X., Zhang Y., Guo Z. (2016). CHIR99021 enhances Klf4 Expression through beta-Catenin Signaling and miR-7a Regulation in J1 Mouse Embryonic Stem Cells. PLoS ONE.

[13] Bain J., Plater L., Elliott M., Shpiro N., Hastie C.J., McLauchlan H., Klevernic I., Arthur J.S., Alessi D.R., Cohen P. (2007). The selectivity of protein kinase inhibitors: A further update. Biochem. J..

[14] Kim S.H., Kim M.O., Cho Y.Y., Yao K., Kim D.J., Jeong C.H., Yu D.H., Bae K.B., Cho E.J., Jung S.K. (2014). ERK1 phosphorylates Nanog to regulate protein stability and stem cell self-renewal. Stem Cell Res..

[15] Kunath T., Saba-El-Leil M.K., Almousailleakh M., Wray J., Meloche S., Smith A. (2007). FGF stimulation of the Erk1/2 signalling cascade triggers transition of pluripotent embryonic stem cells from self-renewal to lineage commitment. Development.

[16] Ai Z., Shao J., Shi X., Yu M., Wu Y., Du J., Zhang Y., Guo Z. (2016). Maintenance of Self-Renewal and Pluripotency in J1 Mouse Embryonic Stem Cells through Regulating Transcription Factor and MicroRNA Expression Induced by PD0325901. Stem Cells Int..

[17] Sun X., Lewandoski M., Meyers E.N., Liu Y.-H., Jr R.E.M., Martin G.R. (2000). Conditional inactivation of Fgf4 reveals complexity of signalling during limb bud development. Nat. Genet..

[18] Lanner F., Rossant J. (2010). The role of FGF/Erk signaling in pluripotent cells. Development.

[19] Bost F., Caron L., Marchetti I., Dani C., Le Marchand-Brustel Y., Binetruy B. (2002). Retinoic acid activation of the ERK pathway is required for embryonic stem cell commitment into the adipocyte lineage. Biochem. J..

[20] Miyanari Y., Torres-Padilla M.E. (2012). Control of ground-state pluripotency by allelic regulation of Nanog. Nature.

[21] Burdon T., Stracey C., Chambers I., Nichols J., Smith A. (1999). Suppression of SHP-2 and ERK signalling promotes self-renewal of mouse embryonic stem cells. Dev. Biol..

[22] Griep A.E., Deluca H.F. (1986). Decreased c-myc expression is an early event in retinoic acid-induced differentiation of F9 teratocarcinoma cells. Proc. Natl. Acad. Sci. USA.

[23] Wang R., Liang J., Yu H.M., Liang H., Shi Y.J., Yang H.T. (2008). Retinoic acid maintains self-renewal of murine embryonic stem cells via a feedback mechanism. Differentiation.

[24] Tay Y., Zhang J., Thomson A.M., Lim B., Rigoutsos I. (2008). MicroRNAs to Nanog, Oct4 and Sox2 coding regions modulate embryonic stem cell differentiation. Nature.

[25] Chen H., Shalom-Feuerstein R., Riley J., Zhang S.D., Tucci P., Agostini M., Aberdam D., Knight R.A., Genchi G., Nicotera P. (2010). miR-7 and miR-214 are specifically expressed during neuroblastoma differentiation, cortical development and embryonic stem cells differentiation, and control neurite outgrowth in vitro. Biochem. Biophys. Res. Commun..

[26] Tay Y.M., Tam W.L., Ang Y.S., Gaughwin P.M., Yang H., Wang W., Liu R., George J., Ng H.H., Perera R.J. (2008). MicroRNA-134 modulates the differentiation of mouse embryonic stem cells, where it causes post-transcriptional attenuation of Nanog and LRH1. Stem Cells.

[27] Niu C.S., Yang Y., Cheng C.D. (2013). MiR-134 regulates the proliferation and invasion of glioblastoma cells by reducing Nanog expression. Int. J. Oncol..

[28] Kaspi H., Chapnik E., Levy M. (2013). Brief report: miR-290-295 regulate embryonic stem cell differentiation propensities by repressing Pax6. Stem Cells.

[29] Wu H., Zhao J., Fu B., Yin S., Song C., Zhang J., Zhao S., Zhang Y. (2017). Retinoic acid-induced upregulation of miR-219 promotes the differentiation of embryonic stem cells into neural cells. Cell Death Dis..

[30] Stavridis M.P., Lunn J.S., Collins B.J., Storey K.G. (2007). A discrete period of FGF-induced Erk1/2 signalling is required for vertebrate neural specification. Development.

[31] Pérez-Santos M.A.-R.C.B.L.M. (2013). miR-485 Acts as a Tumor Suppressor by Inhibiting Cell Growth and Migration in Breast Carcinoma T47D Cells. Asian Pac. J. Cancer Prev..

[32] Lou C., Xiao M., Cheng S., Lu X., Jia S., Ren Y., Li Z. (2016). MiR-485-3p and miR-485-5p suppress breast cancer cell metastasis by inhibiting PGC-1alpha expression. Cell Death Dis..

[33] Obinata D., Takada S., Takayama K., Urano T., Ito A., Ashikari D., Fujiwara K., Yamada Y., Murata T., Kumagai J. (2016). Abhydrolase domain containing 2, an androgen target gene, promotes prostate cancer cell proliferation and migration. Eur. J. Cancer.

[34] Mahood T.H., Johar D.R., Iwasiow B.M., Xu W., Keijzer R. (2016). The transcriptome of nitrofen-induced pulmonary hypoplasia in the rat model of congenital diaphragmatic hernia. Pediatric Res..

[35] Yamanoi K., Matsumura N., Murphy S.K., Baba T., Abiko K., Hamanishi J., Yamaguchi K., Koshiyama M., Konishi I., Mandai M. (2016). Suppression of ABHD2, identified through a functional genomics screen, causes anoikis resistance, chemoresistance and poor prognosis in ovarian cancer. Oncotarget.

[36] Manku G., Wang Y., Merkbaoui V., Boisvert A., Ye X., Blonder J., Culty M. (2015). Role of Retinoic Acid and Platelet-Derived Growth Factor Receptor Cross Talk in the Regulation of Neonatal Gonocyte and Embryonal Carcinoma Cell Differentiation. Endocrinology.

[37] Kim M.O., Kim S.H., Cho Y.Y., Nadas J., Jeong C.H., Yao K., Kim D.J., Yu D.H., Keum Y.S., Lee K.Y. (2012). ERK1 and ERK2 regulate embryonic stem cell self-renewal through phosphorylation of Klf4. Nat. Struct. Mol. Biol..

[38] Strickland S., Mahdavi V. (1978). The induction of differentiation in teratocarcinoma stem cells by retinoic acid. Cell.

[39] Shi Y., Hou L., Tang F., Jiang W., Wang P., Ding M., Deng H. (2005). Inducing embryonic stem cells to differentiate into pancreatic beta cells by a novel three-step approach with activin A and all-trans retinoic acid. Stem Cells.

[40] Arceci R.J., King A.A.J., Simon M.C., Orkin S.H., Wilson D.B. (1993). Mouse gata-4: A retinoic acid-inducible gata-binding transcription factor expressed in endodermally derived tissues and heart. Mol. Cell. Biol..

[41] Chuang J.H., Tung L.C., Lin Y. (2015). Neural differentiation from embryonic stem cells in vitro: An overview of the signaling pathways. World J. Stem Cells.

[42] Li L., Sun L., Gao F., Jiang J., Yang Y., Li C., Gu J., Wei Z., Yang A., Lu R. (2010). Stk40 links the pluripotency factor Oct4 to the Erk/MAPK pathway and controls extraembryonic endoderm differentiation. Proc. Natl. Acad. Sci. USA.

[43] Mendoza-Parra M.A., Malysheva V., Mohamed Saleem M.A., Lieb M., Godel A., Gronemeyer H. (2016). Reconstructed cell fate-regulatory programs in stem cells reveal hierarchies and key factors of neurogenesis. Genome Res..

[44] Li C., Liu B., Zhong S., Wang H. (2016). MEK inhibitor PD0325901 and vitamin C synergistically induce hypomethylation of mouse embryonic stem cells. Oncotarget.

[45] Huang H., Xie C., Sun X., Ritchie R.P., Zhang J., Chen Y.E. (2010). Mir-10a contributes to retinoid acid-induced smooth muscle cell differentiation. J. Biol. Chem..

[46] Glaser T., de Oliveira S.L., Cheffer A., Beco R., Martins P., Fornazari M., Lameu C., Junior H.M., Coutinho-Silva R., Ulrich H. (2014). Modulation of mouse embryonic stem cell proliferation and neural differentiation by the p2x7 receptor. PLoS ONE.

[47] (2012). Esrrb is a direct Nanog target gene that can substitute for Nanog function in pluripotent cells. Cell Stem Cell.

[48] Mitsui K., Tokuzawa Y., Itoh H., Segawa K., Murakami M., Takahashi K., Maruyama M., Maeda M., Yamanaka S. (2003). The homeoprotein Nanog is required for maintenance of pluripotency in mouse epiblast and ES cells. Cell.

[49] Hart A.H., Hartley L., Ibrahim M., Robb L. (2004). Identification, cloning and expression analysis of the pluripotency promoting Nanog genes in mouse and human. Dev. Dyn. Off. Publ. Am. Assoc. Anat..

[50] Li Y., McClintick J., Zhong L., Edenberg H.J., Yoder M.C., Chan R.J. (2005). Murine embryonic stem cell differentiation is promoted by socs-3 and inhibited by the zinc finger transcription factor klf4. Blood.

[51] Wei Z., Gao F., Kim S., Yang H., Lyu J., An W., Wang K., Lu W. (2013). Klf4 organizes long-range chromosomal interactions with the oct4 locus in reprogramming and pluripotency. Cell Stem Cell.

[52] Chen B., Xue Z., Yang G., Shi B., Yang B., Yan Y., Wang X., Han D., Huang Y., Dong W. (2013). Akt-signal integration is involved in the differentiation of embryonal carcinoma cells. PLoS ONE.

[53] Zhou Q., Hong Y., Zhan Q., Shen Y., Liu Z. (2009). Role for kruppel-like factor 4 in determining the outcome of p53 response to dna damage. Cancer Res..

[54] Chen S., Guttridge D.C., You Z., Zhang Z., Fribley A., Mayo M.W., Wang C.Y. (2001). Wnt-1 signaling inhibits apoptosis by activating β-catenin/T cell factor–mediated transcription. J. Cell Biol..

[55] Almeida M., Han L., Bellido T., Manolagas S.C., Kousteni S. (2005). Wnt proteins prevent apoptosis of both uncommitted osteoblast progenitors and differentiated osteoblasts by β-catenin-dependent and-independent signaling cascades involving Src/ERK and phosphatidylinositol 3-kinase/AKT. J. Biol. Chem..

[56] Zhang X.N., Wang C.C., Zhou J. (2018). The long noncoding rna neat1 contributes to hepatocellular carcinoma development by sponging mir-485 and enhancing the expression of the stat3. J. Cell. Physiol..

