# Supplementary material for: Retinoic Acid Induces Differentiation of Mouse F9 Embryonic Carcinoma Cell by Modulating the miR-485 Targeting of Abhd2
Source: Int J Mol Sci. 2019 Apr 26;20(9):2071. doi: 10.3390/ijms20092071 (PMC6539702; doi:10.3390/ijms20092071)
Supplement: Supplementary file 1 [file ijms-20-02071-s001.pdf]

International Journal of *Molecular Sciences*

MDPI

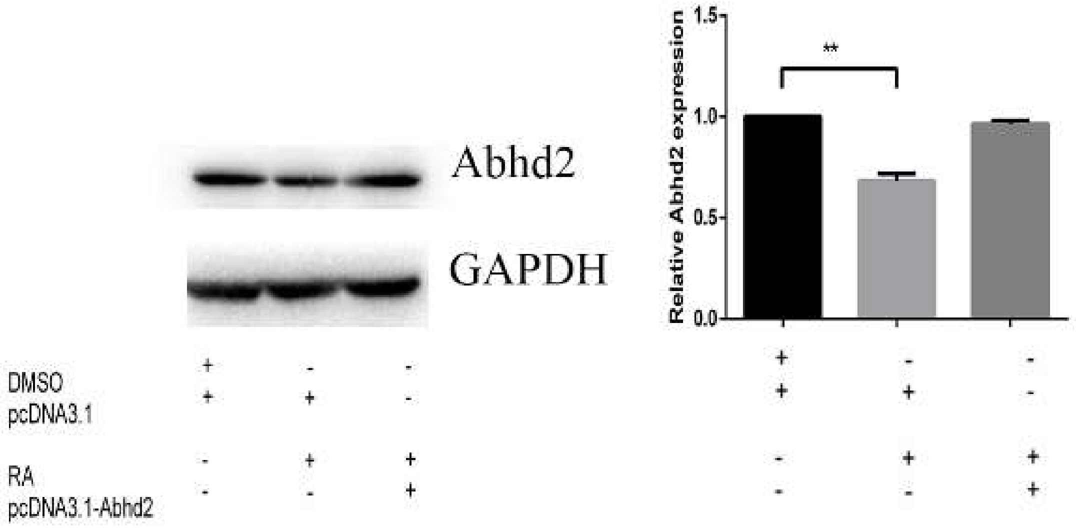

Figure S1. Western Blot analysis with statistical analysis of Abhd2 that were modulated by DMSO, RA or RA with Abhd2. After 6 h transfection, F9 ECs with the pCDNA3.1 or pCDNA3.1-Abhd2 were culture in DMSO or RA contain medium for an additional 30 h. Gapdh was used to normalize template levels. Data are presented as the mean ± SD of three independent experiments (* $p < 0.05$; ** $p < 0.01$; *** $p < 0.001$).

Table S1. Primers used for qPCR analysis.

Gene Name		Primer Sequences (5′ to 3′)
Gapdh	Forward	TGTGAGGGAGATGCTCAGTG
	Reverse	TGTTCCTACCCCCAATGTGT
Nanog	Forward	CACCCACCCATGCTAGTCTT
	Reverse	ACCCTCAAACTCCTGGTCCT
Klf4	Forward	GGCGAGTCTGACATGGCTG
	Reverse	GCTGGACGCAGTGTCTTCTC
Abhd2	Forward	ATGAATGCCATGCTAGAGACCC
	Reverse	GTTCAAACACCGCACGATGA
Pou6f1	Forward	GTCAGATCCTCACGAATGCTC
	Reverse	GAGTCACGGCTTGGACCTG
Tnrc6b	Forward	GCATGGCAAGACTCCACCTC
	Reverse	ACTTGGTTTCGTCACTTCGGG

Table S2. The primer of 3′-UTRs of genes cloned into psiCHECK-2 Vector. (forward primers contains a *Xho*I restriction site and reverse primers contains a *Not*I restriction site, all restriction site letters are underline, mutant primiers contain no restriction site).

Gene Name		Primer Sequences (5′ to 3′)
Abhd2	Forward	CCGCTCGAGACCAAGAAGTCAGCATCCC
	Reverse	ATAAGAATGCGGCCGC TGCCATCTACCTGGTCCAAC
Mut-abhd2	Forward	ACTTGTCTAAAAATTACTAAGACC
	Reverse	GGTCTTAGTAATTTTTAGACAAGT
Dullard	Forward	CCGCTCGAGTTCTCCCAATGCTGGATGCC
	Reverse	ATAAGAATGCGGCCGCAAATGAGGAAAAGTAAGGGGGG
Esrrb	Forward	CCGCTCGAGGCCCCTGCTGAGGCAGACA

	Reverse	ATAAGAATGCGGCCGCCCCACCGTGACACCAACACC
Mut-esrrb	Forward	CAGCCACACTAAGAGACTTTTTCA
	Reverse	TGAAAAAAGTCTCTTAGTGTGGCTG
Pou6f1	Forward	CCGCTCGAGGCTGGGCATGTGGAAGGAGGGGGTT
	Reverse	ATAAGAATGCGGCCGCTGGGTAAGGGCACGAGAGGGGGG AA
Mut-pou6f1	Forward	AACTGACTAAGAACACTGCCTCCCT
	Reverse	AGGGAGGCAGTGTTCTTAGTCAGTT
Sox5	Forward	CCGCTCGAGTTACGAGGAGTACGATGAGG
	Reverse	ATAAGAATGCGGCCGCGAGGAGCAAACCCAGAACCA
Tnrc6b	Forward	CCGCTCGAGATGGAAGTTGTTGCTAAGAA
	Reverse	ATAAGAATGCGGCCGCGAAATAATGAAAAGCGGATG
Mut-tnrc6b	Forward	AAGACTTGACGGCTAAGAACTTTAA
	Reverse	TTAAAGTTCTTAGCCGTCAAGTCTT
Ttyh2	Forward	CCGCTCGAGCCAGCATGAGACCCACCTA
	Reverse	ATAAGAATGCGGCCGCGCTGTCCTTCTGGCATCTAG
tub	Forward	CCGCTCGAGAAGGTTGAACAGGCTGATTT
	Reverse	ATAAGAATGCGGCCGCAACTGGTTTGGAGCAGAGG
Zbtb39	Forward	CCGCTCGAGTGACTTTGTAAAGGCCGTTGG
	Reverse	ATAAGAATGCGGCCGCGGAAATGAGGAGAAAGGTGGAG
Mut-Zbtb39	Forward	GACCAAACTAAGTGCTCGATGGGGT
	Reverse	ACCCCATCGAGCACTTAGTTTGGTC

Table S3. sequence of si-Abhd2 and mimic.

Abhd2-mus-345	GCUCAAAUCCUGUCCUCUUTT	AAGAGGACAGGAUUUGAGCTT
Abhd2-mus-1175	GGAUAUAUGUGCCUCUCAUTT	AUGAGAGGCACAUAUAUCCTT
Abhd2-mus-941	GGUUCUACAACUUCCUCAUTT	AUGAGGAAGUUGUAGAACCTT
mmu-mir-485-5p mimics	AGAGGCUGGCCGUGAUGAAUUC	AUUCAUCACGGCCAGCCUCUUU
mmu-mir-485-5p inhibitor	GAAUUCAUCACGGCCAGCCUCU	
